# Supplementary material for: Splice-Junction-Based Mapping of Alternative Isoforms in the Human Proteome
Source: Cell Rep. Author manuscript; Available in PMC 2020 Jan 15. (PMC6961840; doi:10.1016/j.celrep.2019.11.026)
Supplement: 3 [file NIHMS1546469-supplement-3.zip › DF2/PXD0010154/Prostate-207-P21333-FGGEHVPNSPFQVTAPERPLVGVNGLDVTSLRPFDLVIPFTIK.pdf]

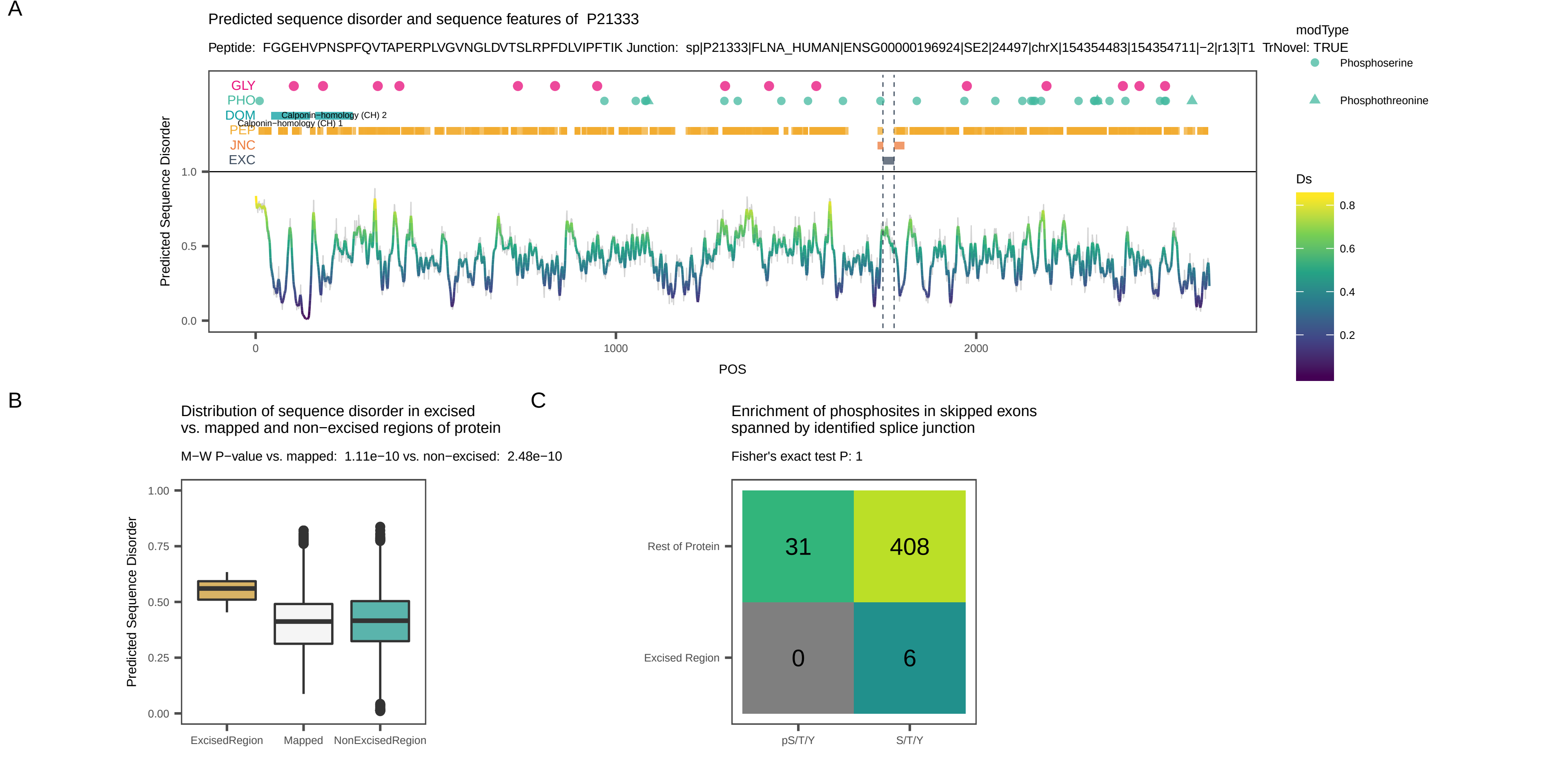
A
Predicted sequence disorder and sequence features of P21333
Peptide: FGGEHVPNSPFQVTAPERPLVGVNGLDVTSLRPFDLVIPFTIK Junction: sp|P21333|FLNA_HUMAN|ENSG00000196924|SE2|24497|chrX|154354483|154354711|−2|r13|T1 TrNovel: TRUE
GLY
PHO
DOM
PEP
JNC
EXC
Calponin−homology (CH) 2
Calponin−homology (CH) 1
Predicted Sequence Disorder
1.0
0.5
0.0
0
1000
2000
POS
modType
Phosphoserine
Phosphothreonine
Ds
0.8
0.6
0.4
0.2
B
Distribution of sequence disorder in excised
vs. mapped and non−excised regions of protein
M−W P−value vs. mapped: 1.11e−10 vs. non−excised: 2.48e−10
Predicted Sequence Disorder
1.00
0.75
0.50
0.25
0.00
ExcisedRegion
Mapped
NonExcisedRegion
C
Enrichment of phosphosites in skipped exons
spanned by identified splice junction
Fisher's exact test P: 1
Rest of Protein
Excised Region
31
408
0
6
pS/T/Y
S/T/Y
